# Effects of living alone versus with others and of housemate type on smoking, drinking, dietary habits, and physical activity among elderly people

**DOI:** 10.4178/epih.e2017034

**Published:** 2017-08-06

**Authors:** Seungmin Jeong, Sung il Cho

**Affiliations:** Graduate School of Public Health, Seoul National University, Seoul, Korea

**Keywords:** Aged, Family characteristics, Health behavior, Korea

## Abstract

**OBJECTIVES:**

This study examined differences in health behaviors between elderly people living alone and with others; it also investigated whether the effect of living with others differs according to housemate type, namely a spouse and/or younger generations.

**METHODS:**

Gender-stratified data from the 2013 Korea Community Health Survey for individuals aged 60 to 74 living in Seoul were analyzed. Logistic regression modeling was conducted to obtain odds ratios (ORs) and 95% confidence intervals (CIs) of the outcome variables (smoking, drinking, eating salty foods, inactive lifestyle) for the variables of interest (living alone/with others, housemate type). Models were adjusted for confounding variables including history of medical conditions, employment type, and adjusted household income.

**RESULTS:**

Analysis involved 1,814 men and 2,199 women. Risk of smoking was 1.80 times (95% CI, 1.21 to 2.67) higher for men living alone than living with others. Risk of eating salty foods was 0.78 times lower (95% CI, 0.62 to 0.98) for men living with a spouse than a spouse and younger generations. Risk of inactive lifestyle was 1.47 times higher (95% CI, 1.13 to 1.92) for women living alone. Risk of smoking was higher for women living alone (OR, 1.41; 95% CI, 1.03 to 1.92) or with younger generations (OR, 9.12; 95% CI, 2.04 to 40.80) than with a spouse and younger generations.

**CONCLUSIONS:**

Living alone was associated with smoking in men and physical activity in women; housemate type was associated with dietary habits in men and smoking in women. These gender-specific findings can help identify groups of individuals vulnerable to risky health behaviors and to develop policies.

## INTRODUCTION

Korea is fast becoming an aged society due to prolonged lifespan thanks to developments in medicine and persistent decrease in birth rate [1]. According to statistics on the elderly population in Statistics Korea 2016, the life expectancy of a 65-year-old person in 2014 was estimated to be 18.3 years in men and 22.8 years in women; after subtracting duration of disease, life expectancy was 8.9 and 9.2 years for men and women, respectively. In 2013, medical expenses for elderly individuals constituted 34.5% of overall medical expenses. Thus, preparations for an aged society should be made in many fields, with public health policy desperately needed.

In the past agrarian society, people traditionally lived with extended family of three or more generations, respecting and assisting elderly individuals. Through urbanization, however, families have become nuclear, and the number of elderly individuals who do not want to depend on their children has increased. Accordingly, the number of elderly individuals living alone or with only a spouse is gradually increasing.

Many research articles have shown that elderly individuals’ health behaviors and health outcomes differ depending on whether they live alone or with others, and if the latter, with whom they live. In particular, many studies have demonstrated that living alone negatively affects health behaviors and health outcomes in the elderly population. Most of these studies examined whether the elderly individuals had a spouse, and some found that married elderly individuals performed more health behaviors than did elderly individuals living alone [2-4]. A recent study investigated the relationship between housemate type, smoking, and drinking, and reported that elderly individuals living with their offspring and a spouse smoked and drank less than those living alone [5].

This study examined whether differences existed in health behaviors such as smoking, drinking, and eating salty foods between elderly individuals living with others and those living alone, and further investigated whether the effect of living with others differed according to housemate type (a spouse or younger generations, such as children or grandchildren). To achieve these objectives, we utilized data from the Korea Community Health Survey (KCHS).

## MATERIALS AND METHODS

### Data source

Data from the 2013 KCHS were analyzed. The KCHS has been conducted since 2008 to generate health-related statistics at the city, county, and district levels with respect to regional public health planning, and to provide basic data for systematic evaluation of regional public health project outcomes. The survey is conducted by 253 public health centers and 35 universities nationwide, all of which are under contract with the Ministry of Health and Welfare and the Korea Centers for Disease Control and Prevention. Survey participants are adults aged 19 or older, and the mean number of participants is 900 per public health center [6]. The following areas are excluded from the household sampling scheme: residential areas in which it is impossible to live due to urban renewal or redevelopment; dense commercial or factory districts where the population is very sparse; and areas where particular groups of people live (e.g., villages for leprosy patients, dormitories, and communal living places for religious group members). Additionally, considering that the survey is conducted via one-on-one inperson interviews, the following cases are excluded: individuals who cannot be contacted after 3 or more attempts, households without an adult aged 19 or older, and household members who cannot be contacted during the survey period. The survey is administered by trained survey administrators who visit the households selected for the survey sample to conduct one-on-one interviews using a computer program. The survey includes items focusing on the individual’s health behavior, healthcare use, quality of life, and so on, as well as items focusing on the household, such as household income and type. Several public health studies based on the KCHS data have been published, and further details about the survey can be found in previously published articles [6-8].

### Selection of study subjects

We reasoned that gender, area of residence, and age would have the greatest influence as confounding variables. Hence, study subjects were selected by limiting area of residence to Seoul and age to the range between 60 and 74. Data were stratified by gender and analyzed separately for men and women.

### Variable measurements

Based on responses to the items regarding household type and whether the respondent lived with a spouse, all respondents were classified into the categories of elderly individuals living alone, elderly couples, elderly individuals living with younger generations without a spouse, or elderly individuals living with younger generations and a spouse. Elderly couples included not only couples in a marriage, but also those in a cohabitation without the wedding. Hypertension, diabetes mellitus, myocardial infarction, and stroke were considered only if diagnosed by a physician. Adjusted monthly household income was based on a household equivalence scale, with the value obtained by dividing monthly household income by the square root of household size [9]. Drinking was categorized into 3 groups based on the amount of alcohol consumed at once, according to the World Health Organization definition: no drinking, moderate drinking (pure alcohol of 0.10 to 19.99 g for women and 0.10 to 39.99 g for men), and excessive drinking (pure alcohol ≥ 20 g for women and ≥ 40 g for men) [10]. The dietary habit of eating salty foods was categorized based on the subject’s response to the item, “Which of the following descriptions is most appropriate for you when you eat?” Physical activity (PA) was categorized as follows: inactive, if the respondent never performed high-impact or moderate-impact PAs; active, if the respondent performed high-impact PAs for 20 minutes at least 3 times weekly, or moderate-impact PAs for 30 minutes at least 5 times weekly; and less active, if the respondent exercised regularly, but not at the level defined as “active.”

### Statistical analysis

Demographic characteristics are described by gender. Multivariate logistic regression modeling was performed to compute the odds ratios (ORs) and 95% confidence intervals (CIs) for the variables of major interest (i.e., living alone vs. with others, and type of housemates). The outcome variables of the logistic modeling were smoking, excessive drinking, eating salty foods, and inactive lifestyle. Dietary habits were categorized into 3 groups when transformed into a categorical variable, but grouped into 2 in the logistic regression analysis (i.e., eating salty foods and not eating salty foods). PA was also categorized into 2 groups in the logistic regression analysis (i.e., inactive and active). Confounding variables included in the regression analysis for adjustment were age (60-64, 65-69, and 70-74); history of hypertension, diabetes mellitus, myocardial infarction, and stroke; employment type; and adjusted monthly household income. When used to adjust the regression analysis, employment type was categorized into 3 groups: 1) employer or business owner; 2) wage earner; and 3) no job or unpaid family worker. Household income per person was grouped into under 2 million won and over 2 million won. Data were analyzed using SAS version 9.4 (SAS Institute, Inc., Cary, NC, USA).

## RESULTS

### Demographic characteristics

A total of 23,139 adults aged 19 or older living in Seoul participated in the 2013 KCHS; of those, 4,438 were elderly individuals 60 and 74. A total of 4,013 subjects were included in the final analysis, after excluding 425 because their living status (alone or otherwise) could not be confirmed, they did not meet the classification criteria of the present study, or their information was not confirmed. A total of 1,814 men and 2,199 women were included in the final analysis, and their data were separately analyzed.

Demographic characteristics of the total study sample are shown in Table 1. The proportion of elderly individuals living alone was higher among women (18.4%) than among men (7.1%). Regarding employment type, the proportion of individuals with no job varied between genders: the proportion was higher in elderly men living alone, but lower in the corresponding group of women. In both men and women, adjusted monthly household income was lower in elderly individuals living alone or with only a spouse compared to other groups. The proportion of smokers was higher in both elderly men and elderly women living alone.

### Major analytic results

High-risk health behaviors were defined as smoking, excessive drinking, eating salty foods, and inactive lifestyle. To investigate the effects on high-risk health behaviors of living alone vs. with others, ORs adjusted for confounding variables and 95% CIs were computed. Similar analysis was performed to investigate the effects of housemate type. The Hosmer-Lemeshow goodness-of-fit test confirmed that all models were fit.

Among men, the risk of smoking was 1.80 times higher (95% CI, 1.21 to 2.67) for those living alone than those living with others. Analysis of housemate type showed that the risk of eating salty foods was 0.78 times lower (95% CI, 0.62 to 0.98) for those living with only a spouse compared to those living with a spouse and younger generations (95% CI, 0.62 to 0.98) (Table 2).

Among women, the risk of inactive lifestyle was 1.47 times higher (95% CI, 1.13 to 1.92) for those living alone than those living with others. Analysis of housemate type showed that the risk of smoking was 9.12 times higher (95% CI, 2.04 to 40.80) for those living with only younger generations and 1.41 times higher (95% CI, 1.03 to 1.92) for those living alone compared to those living with a spouse and younger generations (Table 2).

## DISCUSSION

The study showed that the risk of smoking in men and inactive lifestyle in women was higher for elderly individuals living alone, and that the risk of eating salty foods in men and smoking in women differed depending on housemate type.

In a previous study conducted of elderly individuals by Zhang & Wu [5] the risk of smoking was found to be lower for men living with children and for women living with children or with both children and a spouse. In both genders, the risk of drinking was found to be lower for elderly individuals living with children and a spouse than for those living alone. The present study’s findings also show that the risk of smoking was lower for men living with others and for women living with younger generations and a spouse, but that it was rather higher for women living with only younger generations. Furthermore, drinking was not found to be associated with either living alone or housemate type. A previous study indicated that living with family influences healthy habits through direct and indirect social control [11]. Direct social control refers to changing healthy habits due to persuasion and requests from people around the individual, and indirect social control refers to making an effort to form healthier habits because of responsibility for the family. To summarize the previous and present study findings in combination, in men’s smoking behaviors, the influence of direct and indirect social control from the people they live with seems critical. For women, smoking has long been perceived as inappropriate by the current generation of elderly individuals [12]. It is believed that women who smoke in such a society have a stronger affinity for smoking than male smokers, and that the effect of social control is shown when it is exerted by more people. Furthermore, elderly individuals have fewer parental responsibilities and duties than younger parents, as their children have already grown; thus, they are less influenced by indirect social control based on their role as a parent. Accordingly, the risk of smoking can be high even for women living with younger generations, as found in the present study [13]. Unlike the study by Zhang & Wu [5], the present study did not find significant relationships between drinking and living alone or housemate type in either men or women. The differences in these findings may be the result of a cultural difference in the research environment.

Compared to the study by Zhang & Wu [5], the present study additionally investigated the relationships of eating salty foods and physical activity. The risk of inactive lifestyle was significantly higher in elderly women living alone. A previous study reported that the proportion of elderly women who indicated a lack of company as a hindrance to performing physical activity was twice that of elderly men [14]. Considering that in most cases the exercise companions of an elderly individual are family and friends [15], elderly women living alone are less likely than men to perform physical activity, due to a lack of company.

Interestingly, a statistically significant relationship between eating salty foods less and living with a spouse was found in men only. This finding may be due to the characteristic of Korean society that elderly men rarely cook, instead eating foods cooked by other members of the family. Thus, elderly men living with a spouse typically eat foods cooked by their spouse, and those living with other types of housemate eat foods cooked by younger members of the family. Statistical analysis of the data source showed that the proportion of people eating salty foods was lower in elderly women between 60 and 74 compared to adults between 19 and 59 (22.4 vs. 27.0%, p< 0.01). In the case of women, they cook if living with a spouse, and are highly likely to cook even if they live with younger generations. Thus, it can be reasoned that there was no difference in women according to household type, unlike in men.

Based on the above discussion, the effects of living alone vs. with others and of housemate type on health behaviors differ depending on gender, and these differences seem to be due to the process through which gender-specific lifestyle habits develop. Therefore, these points should be reflected in policy developed to improve the health behaviors of elderly individuals. An approach utilizing direct social control, such as education, persuasion, and requests, can be considered for elderly men living alone who have a high risk of smoking. For women living alone, who are at high risk of inactive lifestyle, it may be useful to help them acquire an exercise companion or provide them with community group exercise services.

There are a few limitations affecting the present study. The first is a possibility of selection bias. Because the KCHS is administered via one-on-one in-person interviews, individuals who cannot be contacted after 3 or more attempts, or who strongly decline the survey, are not included. Thus, it is possible that individuals not surveyed may have poorer health behavior. Second, regarding the outcome variables, eating salty foods was based not on an objective measurement but on subjective judgment, and it is thus possible that the measurement was inaccurate. However, we believe the difference between subjective judgment and actual intake of salty foods is not significant. Third, because the confounding variables of the present study (i.e., hypertension, diabetes mellitus, myocardial infarction, and stroke) are indicators of health status, they may be secondary outcome variables derived from the outcome variable of the study (i.e., health behavior). However, we determined that these factors could not be omitted because of their important influence on health behavior, and we thus included them as confounding variables based on the understanding that their roles in the current analysis were greater as causes rather than outcomes of health behavior. Therefore, the results reported in the present study can be interpreted as results obtained from individuals whose health outcomes with respect to hypertension, diabetes mellitus, stroke, and myocardial infarction are the same. Finally, like previous studies, the present study did not examine the possibility that not only the presence of housemates but also the characteristics of their health behavior influence elderly individuals. Additional research is needed to address the issue.

In conclusion, in both genders, the risk of smoking was higher for elderly individuals living alone compared to those living with a spouse and younger generations. In elderly women specifically, the risk of smoking was also higher for those living with younger generations without a spouse. The risk of inactive lifestyle was higher for elderly women living alone. In men, the risk of eating salty foods was lower for those living with only a spouse in comparison to those living with a spouse and younger generations. However, there was no significant difference in drinking according to living alone vs. living with others, or according to type of housemates. Based on the findings and analysis, policies may be developed that can aggressively influence elderly individuals to change their lifestyle habits to improve their health behavior, which is found to be gender-specific.

## Figures and Tables

**Table 1.** Demographic characteristics of the study population

	Men					Women				
	Total	Living alone	Living together with			Total	Living alone	Living together with		
			Spouse only	Younger generation only	Spouse and younger generation			Spouse only	Younger generation only	Spouse and younger generation
Total		128(7.1)	872 (48.1)	69 (3.8)	745 (41.1)		404(18.4)	862 (39.2)	373(17.0)	560 (25.5)
Age (yr)										
60-64	643 (35.4)	45 (35.2)	225 (25.8)	26 (37.7)	347 (25.8)	875 (39.8)	125 (30.9)	324 (37.6)	133 (35.7)	293 (52.3)
65-69	607 (33.5)	42 (32.8)	317(36.4)	20 (29.0)	228 (36.4)	689 (31.3)	133 (32.9)	294 (34.1)	89 (23.9)	173 (30.9)
70-74	564 (31.1)	41 (32.0)	330 (37.8)	23 (33.3)	170 (37.8)	635 (28.9)	146(36.1)	244 (28.3)	151 (40.5)	94(16.8)
Chronic disease										
Hypertension	873 (48.1)	71 (55.5)	425 (48.7)	27(39.1)	350 (48.7)	1034 (47.0)	199 (49.3)	395 (45.8)	191 (51.2)	249 (44.5)
Diabetes mellitus	386 (21.3)	38 (29.7)	180 (20.6)	18(26.1)	150 (20.6)	401 (18.2)	77(19.1)	156(18.1)	88 (23.6)	80(14.3)
Myocardial infarction	77 (4.2)	5 (3.9)	40 (4.6)	5 (7.2)	27 (4.6)	37(1.7)	7(1.7)	10(1.2)	11 (2.9)	9(1.6)
Stroke	90 (5.0)	11 (8.6)	43 (4.9)	1 (1-4)	35 (4.9)	72 (3.3)	15(3.7)	28 (3.2)	18(4.8)	11 (2.0)
Employment status										
Self-employed/employer	419(23.1)	18(14.1)	195 (22.4)	15(21.7)	191 (22.4)	134 (6.1)	24 (5.9)	43 (5.0)	31 (83)	36 (6.4)
Employee	488 (26.9)	31 (24.2)	185 (21.2)	15(21.7)	257(21.2)	378(17.2)	96 (23.8)	119(13.8)	62(16.6)	101 (18.0)
Unpaid family worker	5 (0.3)	0 (0.0)	1 (0.1)	0 (0.0)	4(0.1)	31 (1.4)	2 (0.5)	15(1.7)	1 (0.3)	13(2.3)
Unemployed	902 (49.7)	79 (61.7)	491 (56.3)	39 (56.5)	293 (56.3)	1656 (75.3)	282 (69.8)	685 (79.5)	279 (74.8)	410(73.2)
Adjusted monthly household income (won)										
<1,000,000	529 (29.2)	65 (50.8)	328 (37.6)	11 (15.9)	1 25 (37.6)	800 (36.4)	229 (56.7)	377 (43.7)	70(18.8)	124(22.1)
1,000,000-2,000,000	540 (29.8)	22(17.2)	270 (31.0)	22(31.9)	226 (31.0)	650 (29.6)	97 (24.0)	255 (29.6)	138 (37.0)	160(28.6)
2,000,000-3,000,000	318(17.5)	10(7.8)	114(13.1)	16(23.2)	178(13.1)	322(14.6)	25 (6.2)	96(11.1)	78 (20.9)	123 (22.0)
>3,000,000	358(19.7)	25(19.5)	139(15.9)	15(21.7)	179(15.9)	341 (15.5)	39 (9.7)	112(13.0)	73(19.6)	117(20.9)
Missing	69 (3.8)	6 (4.7)	21 (2.4)	5 (7.2)	37 (2.4)	86 (3.9)	14(3.5)	22 (2.6)	14(3.8)	36 (6.4)
Smoking status										
Smoker	445 (24.5)	46 (35.9)	187 (21.4)	18(26.1)	194 (21.4)	40(1.8)	14(3.5)	9(1.0)	15 (4.0)	2 (0.4)
Ex-smoker	974 (53.7)	60 (46.9)	486 (55.7)	37 (53.6)	391 (55.7)	33(1.5)	13(3.2)	6 (0.7)	9(2.4)	5 (0.9)
Non-smoker	395 (21.8)	22(17.2)	199 (22.8)	14(20.3)	160 (22.8)	2,126 (96.7)	377 (93.3)	847 (98.3)	349 (93.6)	553 (98.8)
Alcohol consumption										
Heavy drinking	570 (31.4)	49 (38.3)	246 (28.2)	22(31.9)	253 (28.2)	232(10.6)	44(10.9)	90(10.4)	43(11.5)	55 (9.8)
Moderate drinking	763 (42.1)	41 (32.0)	389 (44.6)	27(39.1)	306 (44.6)	708 (32.2)	117(29.0)	283 (32.8)	109 (29.2)	199 (35.5)
Non-drinking	481 (26.5)	38 (29.7)	237(27.2)	20 (29.0)	186 (27.2)	1,259 (57.3)	243 (60.1)	489 (56.7)	221 (59.2)	306 (54.6)
Salt habit										
Eat salty	522 (28.8)	41 (32.0)	234 (26.8)	19(27.5)	228 (26.8)	495 (22.5)	94 (23.3)	181 (21.0)	94 (25.2)	126(22.5)
Eat normally	710(39.1)	46 (35.9)	355 (40.7)	28 (40.6)	281 (40.7)	1,059 (48.2)	197 (48.8)	409 (47.4)	178 (47.7)	275 (49.1)
Eat less salty	582 (32.1)	41 (32.0)	283 (32.5)	22 (31.9)	236 (32.5)	645 (29.3)	113(28.0)	272 (31.6)	101 (27.1)	159(28.4)
Physical Activity										
Non activity	999 (55.1)	70 (54.7)	483 (55.4)	40 (58.0)	406 (55.4)	1,526 (69.4)	引 3 (7入5)	566 (65.7)	272 (72.9)	375 (67.0)
Mid-activity	349(19.2)	17(13.3)	171 (19.6)	10(14.5)	151 (19.6)	337(15.3)	42(10.4)	133(15.4)	65(17.4)	97(17.3)
High activity	466 (25.7)	41 (32.0)	218(25.0)	19(27.5)	188 (25.0)	336(15.3)	49(12.1)	163(18.9)	36 (9.7)	88(15.7)

Values are presented as number (%).

**Table 2.** Results of logistic regression analysis for association between health behaviors and type of housemates by gender

	Total	High risk health behavior	Crude OR (95% CI)	Adjusted OR (95% CI)
Men				
Smoking	1814	445		
Living alone	128	46	1.80 (1.22, 2.66)	1.80 (1.21, 2.67)
Living together	1686	399	1.00 (reference)	1.00 (reference)
By type with inmate				
None	128	46	1.62 (1.08, 2.44)	1.61 (1.06, 2.45)
Spouse only	872	187	0.80 (0.64, 1.02)	0.81 (0.63,1.04)
Younger generation only	69	18	1.08 (0.60, 1.92)	1.12 (0.62, 2.01)
Spouse and younger generation	745	194	1.00 (reference)	1.00 (reference)
Heavy drinking	1,814	570		
Living alone	128	49	1.51 (1.04, 2.20)	1.46 (0.99, 2.15)
Living together	1686	521	1.00 (reference)	1.00 (reference)
By type with inmate				
None	128	49	1.33 (0.90, 1.98)	1.38 (0.92, 2.08)
Spouse only	872	246	0.79 (0.63, 0.97)	0.90 (0.71, 1.13)
Younger generation only	69	22	0.97 (0.56, 1.67)	1.09 (0.63,1.91)
Spouse and younger generation	745	253	1.00 (reference)	1.00 (reference)
Salty habit	1814	522		
Living alone	128	41	1.15 (0.77, 1.71)	1.11 (0.75,1.67)
Living together	1686	481	1.00 (reference)	1.00 (reference)
By type with inmate				
None	128	41	1.04 (0.69, 1.57)	0.96 (0.63,1.47)
Spouse only	872	234	0.84 (0.67, 1.05)	0.78 (0.62, 0.98)
Younger generation only	69	19	0.76 (0.43, 1.38)	0.76 (0.42,1.38)
Spouse and younger generation	745	228	1.00 (reference)	1.00 (reference)
No physical activity	1814	999		
Living alone	128	70	1.03 (0.71, 1.49)	0.84 (0.66,1.07)
Living together	1686	929	1.00 (reference)	1.00 (reference)
By type with inmate				
None	128	70	1.06 (0.72, 1.56)	0.85 (0.57,1.27)
Spouse only	872	483	1.06 (0.87, 1.30)	0.85 (0.69,1.05)
Younger generation only	69	40	1.09 (0.65, 1.82)	1.03 (0.61,1.74)
Spouse and younger generation	745	406	1.00 (reference)	1.00 (reference)
Women				
Smoking	2199	40		
Living alone	404	14	2.25 (1.15, 4.42)	1.86 (0.93, 3.73)
Living together	1795	26	1.00 (reference)	1.00 (reference)
By type with inmate				
None	404	14	9.00 (2.02,40.10)	6.11 (1.35, 27.76)
Spouse only	862	9	2.83 (0.61,13.13)	2.11 (0.45, 9.91)
Younger generation only	373	15	11.38 (2.02, 40.10)	9.12 (2.04, 40.80)
Spouse and younger generation	560	2	1.00 (reference)	1.00 (reference)
Heavy drinking	2199	232		
Living alone	404	44	1.06 (0.74,1.50)	1.04 (0.72,1.50)
Living together	1795	188	1.00 (reference)	1.00 (reference)
By type with inmate				
None	404	44	1.13 (0.74,1.72)	1.20 (0.77,1.87)
Spouse only	862	90	1.06 (0.74,1.53)	1.14 (0.79,1.66)
Younger generation only	373	43	1.17 (0.76,1.81)	1.39 (0.89, 2.17)
Spouse and younger generation	560	55	1.00 (reference)	1.00 (reference)
	Total	High risk health behavior	Crude OR (95% CI)	Adjusted OR (95% CI)
Salty habit	2,199	495		
Living alone	404	94	1.01 (0.78, 1.32)	0.90 (0.68,1.17)
Living together	1,795	401	1.00 (reference)	1.00 (reference)
By type with inmate				
None	404	94	1.01 (0.74, 1.39)	0.83 (0.60,1.16)
Spouse only	862	181	0.93 (0.71, 1.21)	0.84 (0.64, 1.11)
Younger generation only	373	94	1.18 (0.86, 1.62)	1.07 (0.78,1.48)
Spouse and younger generation	560	126	1.00 (reference)	1.00 (reference)
No physical activity	2,199	1,526		
Living alone	404	313	167 (1.29, 2.16)	1.47 (1.13,1.92)
Living together	1,795	1,213	1.00 (reference)	1.00 (reference)
By type with inmate				
None	404	313	1.77 (1.31, 2.38)	1.41 (1.03,1.92)
Spouse only	862	566	0.98 (0.78,1.24)	0.84 (0.66,1.07)
Younger generation only	373	272	1.39 (1.04,1.67)	1.25 (0.92,1.70)
Spouse and younger generation	560	375	1.00 (reference)	1.00 (reference)

The adjusted model included potential confounding factors (age, past medical history: hypertension, diabetes mellitus, myocardial infarction, stroke, employment status, household income).

OR, odds ratio; CI, confidence interval.
