# Supplementary material for: Effects of living alone versus with others and of housemate type on smoking, drinking, dietary habits, and physical activity among elderly people
Source: Epidemiol Health. 2017 Aug 6;39:e2017034. doi: 10.4178/epih.e2017034 (PMC5675988; doi:10.4178/epih.e2017034)
Supplement: Supplementary file 1 [file epih-39-e2017034-supplementary.pdf]

Volume: 39, Article ID: e2017034

https://doi.org/10.4178/epih.e2017034

노인의 독거 여부 및 동거인의 유형이 흡연, 음주, 식습관 및 신체활동에 미치는 영향

Effects of living alone versus with others and of housemate type on smoking, drinking, dietary habits, and physical activity among elderly people

정승민, 조성일

서울대학교 보건대학원

교신저자: 조성일, 서울특별시 관악구 관악로1 서울대학교 보건대학원

이메일: scho@snu.ac.kr

Volume: 39, Article ID: e2017034

https://doi.org/10.4178/epih.e2017034

Abstract

Objectives: 이 논문에서는 첫째, 독거노인과 비독거노인간에 흡연, 음주, 짜게 먹는 습관, 신체 활동 등 건강행태에 차이가 있는지에 대해 확인하며, 비독거노인의 경우, 동거인의 유형 따라 그 효과에 차이가 있는지에 대해 탐구하였다.

Methods: 본 연구는 2013년 지역사회건강조사 자료를 이용하였으며 분석 대상은 서울시 만 나이 60세 이상 74세 이하의 노인이었다. 성별에 따라 층화분석하였다. 주요 분석에서는 로지스틱 회귀분석 모델을 통하여 결과변수인 흡연, 과도한 음주, 짜게 먹는 식습관, 신체활동 없음에 대해 주요 관심변수인 독거 유무와 동거인의 종류에 대한 오즈비와 95% 신뢰구간을 구하였다. 고혈압 병력, 당뇨 병력, 심근경색 병력, 뇌졸중 병력, 고용 형태, 보정한 가구 수입을 혼란변수로 보정하여 분석하였다.

Results: 서울시의 60-74세 노인 남성 1,814명, 여성 2,199명을 분석한 결과, 남성에서 독거노인이 비독거노인에 비해 흡연의 위험이 1.80배 높았으며(95% CI 1.21-2.67), 동거인의 종류에 따라 분류하여 분석하였을 때, 남성에서 짜게 먹는 식습관은 배우자와만 함께사는 경우가 배우자 및 젊은 세대와 함께 사는 경우에 비해 0.78배 낮은 것으로 나타났다(95% CI 0.62-0.98). 여성에서 신체활동이 없음의 위험이 독거노인이 비독거노인에 비해 1.47배 높았다(95% CI 1.13-1.92). 동거인의 종류에 따라 분류하여 분석하였을 때, 흡연의 위험이 혼자 사는 노인(OR 1.41, 95% CI 1.03-1.92)과 배우자 없이 젊은 세대와 함께 사는

Volume: 39, Article ID: e2017034

https://doi.org/10.4178/epih.e2017034

노인(OR 9.12, 95% CI 2.04-40.80)이 배우자 및 젊은 세대와 함께 사는 노인에 비해 높았다.

Conclusions: 우리 연구 결과, 독거가 남성의 흡연 및 여성의 신체활동과 연관이 있는 것으로 나타났으며, 동거인의 유형이 남성의 짜게 먹는 습관 및 여성의 흡연과 연관이 있는 것으로 나타났다. 이 결과를 위험건강행태의 취약 계층을 발견하는데 활용할 수 있으며, 결과의 성특이성을 바탕으로 대상에 특화된 정책을 수립하는데 도움을 줄 수 있다.

KEYWORDS: Aged, Family chariateristics, Health behavior

Volume: 39, Article ID: e2017034

https://doi.org/10.4178/epih.e2017034

서론

의학의 발전으로 인한 평균 수명의 증가 및 지속적인 출산률 감소로 인하여 우리 나라도 빠른 속도로 고령화 사회에 진입하고 있다.[1] 2016년 통계청의 고령자 통계에 따르면, 2014년 기준 65세 고령자의 기대여명은 남자 18.3년이고 여자 22.8년이고 유병기간을 제외한 기대여명은 남자 8.9년, 여자 9.2년이다. 2013년 고령자 진료비는 전체 진료비의 34.5%를 차지한다. 이에 사회 여러 분야에서의 고령화 사회에 대한 대비가 필요하며, 그 중에서도 보건정책의 필요성이 절실하다.

이전 농경 사회에서는 전통적으로 3대 이상의 대가족이 함께 살며, 노인을 공경하고 돕는 문화가 있었으나, 도시화를 통하여 핵가족화가 진행하고 자녀에게 신세지지 않으려는 노인이 증가하면서 점차 노인독거와 노인 부부끼리만 사는 경우가 증가하고 있다.

이러한 독거 여부 및 동거자에 따라 노인의 건강 행태 및 건강 결과에 차이가 있다는 여러 기존 논문들이 있었으며, 특히 노인의 독거가 건강 행태 및 건강 결과에 좋지 않은 영향을 미친다는 논문들이 많이 출판되었다. 이러한 논문들은 주로 배우자의 유무를 확인한 연구가 많았는데, 결혼한 상태의 노인이 혼자 사는 노인에 비해 더 좋은 건강 행태를 보인다는 논문이 있었다[2-4]. 최근에는 동거자의 유형과 흡연, 음주의 연관성에 대한 연구가 있었는데, 배우자 외에도 자식과 함께 사는 노인이 혼자 사는 노인에 비해 흡연과 음주를 덜 한다는 보고가 있었다.[5]

Volume: 39, Article ID: e2017034
https://doi.org/10.4178/epih.e2017034

이 논문에서는 지역사회건강조사 자료를 이용하여 비독거노인이 독거노인에 비해 흡연, 음주, 짜게 먹는 습관 등 건강행태에 차이가 있는지에 대해 확인하며, 그 중에서도 동거인이 배우자인지, 자녀, 손자녀와 같은 젊은 세대인지에 따라 그 효과에 차이가 있는지에 대해 탐구하고자 한다.

방법

1. 자료원

본 연구는 2013년 지역사회건강조사 자료를 이용하여 분석하였다.

지역사회 건강조사는 지역 보건의료 계획 수립에 대한 시, 군, 구 단위의 건강 통계를 산출하고 지역보건사업 성과를 체계적으로 평가할 수 있는 기초 자료를 생산하기 위해 보건복지부, 질병관리본부가 2008년부터 전국 253개 보건소와 35개 대학교와 협약을 체결하여 시행해왔다. 지역사회건강조사의 조사대상은 만 19세 이상 성인으로, 보건소별로 평균 900명이다.[6] 재개발 혹은 재건축으로 거주가 불가능한 지역, 상업 혹은 공단 밀집지역으로 거주가 희박한 지역, 특정집단(한센촌, 기숙사, 종교단체집단거주지) 거주지역 등은 표본가구에서 제외한다. 또한 1대1 면접조사임을 감안하여 3회 이상 접촉이 불가능하거나 만 19세 이상 성인이 거주하지 않은 가구는 조사대상에서 제외하고, 조사기간내에 접촉 불가능한 가구원도 조사대상에서 제외한다. 지역사회건강조사는 표본으로 선정된 가구에 훈련된 조사원이 방문하여 컴퓨터 프로그램을 이용하여 1대1 면접조사로 진

Volume: 39, Article ID: e2017034

https://doi.org/10.4178/epih.e2017034

행한다. 문항은 건강행태, 의료이용, 삶의 질 등에 관한 개인조사와 가구소득, 거주형태에 관한 가구조사로 구성되어 있다. 보건학 분야에서 지역사회건강조사 자료를 이용한 여러 논문이 출판되었으며, 조사에 대한 기타 자세한 사항은 기존에 출판된 논문 자료물에서 찾아볼 수 있다.[6-8]

2. 연구대상선정

우리는 이 연구에서 성별과 거주지역, 연령이 혼란변수로서 가장 큰 영향을 미칠 것으로 생각하였다. 그리하여 거주지역은 서울시내로 제한하였고, 연령은 만 나이 60세 이상 74세 이하로 제한하여 연구대상을 선정하였다. 성별에 대해서는 층화하여 따로 분석하였다.

3. 변수 측정 방법

본인이 속한 가구의 유형과 배우자와 함께 사는지에 대해 질문하는 문항에 대한 대답을 바탕으로 대상을 독거노인, 노인 부부, 배우자 없이 젊은 세대와 함께 사는 노인, 배우자와 젊은 세대와 함께 사는 노인으로 구분하였다. 노인 부부는 법적인 혼인 상태 뿐만 아니라 사실혼 관계도 포함하였다. 고혈압, 당뇨, 심근경색증, 뇌졸중은 의사에게 진단받은 적이 있는 경우에만 해당하는 것으로 하였다. 보정한 월 가구 소득은 월 가구소득을 가구 구성원의 등가규모로서 제곱근값을 구하여 나눈 값으로 하였다.[9] 음주는 WHO 정의에 따라, 한 번에 마시는 술의 양을 기준으로 음주하지 않음, 적당한 음주 (여성, 순수 알코올 0.1-19.99 g; 남성 순수 알코올 0.1-39.99 g), 과도한 음주 (여성, 순수 알코올 ≥20 g;

남성 순수 알코올 ≥40 g)의 세 가지로 분류하였다.[10] 짜게 먹는 식습관은 "평상시 음식을 먹을 때, 다음 중 본인은 어디에 해당합니까"라는 질문에 대한 응답으로 구분하였다. 신체활동은 고강도 또는 중등도 신체활동을 전혀 하지 않는다고 대답한 경우, "신체활동 없음"이라고 하였고, 일주일에 20분 이상의 고강도 신체활동을 3회 이상하거나, 30분 이상의 중강도 신체활동을 5회 이상하는 경우를 "활발한 신체활동"라고 정의하였으며, 정기적인 운동을 하지만 "활발한 신체활동"의 정의에 미치지 못하는 경우를 "중간 신체활동"으로 정의하였다.

4. 통계적 분석

성별에 따라 인구학적 특성을 서술하였다. 다변수 로지스틱 회귀분석 모델을 통하여 결과변수인 흡연, 과도한 음주, 짜게 먹는 식습관, 신체활동 없음에 대해 주요 관심변수인 동거 여부와 동거인의 종류에 대한 오즈비와 95% 신뢰구간을 구하였다. 식습관을 범주형 변수로 나눌 때에는 3단계로 분류하였으나, 로지스틱 회귀분석에서는 짜게 먹는다고 대답한 경우와 그렇지 않은 경우 두 가지로 나누었다. 신체활동에 대해서도 로지스틱 회귀분석에서는 "신체활동 없음"와 "신체활동 있음"으로 분류하였다. 연령(60-64세, 65-69세, 70-74세), 고혈압 병력, 당뇨 병력, 심근경색 병력, 뇌졸중 병력, 고용 형태, 보정한 월 가구 소득을 혼란변수로 두고, 회귀분석의 보정에 포함시켰다. 회귀분석 보정에 사용할 때에는 고용 형태는 1)고용주, 개인사업, 2) 임금노동자, 3) 직업 없음 및 무급가족 봉사자로

Volume: 39, Article ID: e2017034

https://doi.org/10.4178/epih.e2017034

분류했으며, 가정내 성인 1인당 수입은 200만원 미만과 200만원 이상으로 분류하였다. SAS software, version 9.4 (SAS Institute Inc., Cary, NC, USA)를 이용하여 분석을 시행하였다.

결과

1. 인구학적 특성

2013년 서울시 19세 이상 성인 23,139명이 지역사회 건강조사에 참여하였다. 이 중 4,438명이 60-74세 노인이었으며, 독거 여부를 확인할 수 없거나 우리 연구 분류 기준에 해당하지 않거나 확인할 수 없는 425명을 제외한 4,013명이 총 연구 대상이 되었다. 이 중 남성 1,814명, 여성 2,199명을 각각 따로 분석하였다.

전체 연구대상 인구집단의 인구학적 특성을 Table1에 제시하였다. 독거노인의 비율은 남성(7.1%)에 비해 여성(18.4%)에서 더 높았다. 고용형태에서 직업없음의 비율의 경우 성별에 따른 차이가 있었다. 남성은 독거노인 그룹에서 높았으나, 여성에서는 독거노인 그룹에서 낮은 비율을 보였다. 남녀모두 보정한 월 가구 소득은 독거노인, 비독거 노인 중 배우자와만 함께 사는 노인이 다른 그룹에 비해 낮았다. 남녀 모두 흡연의 비율이 독거노인에서 높았다.

2. 주요 분석 결과

현재 흡연, 과도한 음주, 짜게 먹는 식이 습관, 신체 활동 없음을 고위험 건강습관으로

https://doi.org/10.4178/epih.e2017034

두고, 독거 여부가 이와 같은 건강 행태에 미치는 효과에 대해 혼란변수를 보정한 오즈비와 95% 신뢰구간을 구하였다. 동거인의 종류에 따라서도 같은 분석을 시행하였다. The Hosmer-Lemeshow Goodness-of-Fit Test에서 로짓 모형들은 모두 적합한 것으로 나타났다

남성에서 독거노인이 비독거노인에 비해 흡연의 위험이 1.80배 높았다 (95% CI 1.21-2.67). 동거인의 종류에 따라 분류하여 분석하였을 때, 남성에서 짜게 먹는 식습관은 배우자와만 함께 사는 경우가 배우자 및 젊은 세대와 함께 사는 경우에 비해 0.78배 낮은 것으로 나타났다(95% CI 0.62-0.98) (Table 2).

여성에서는 신체활동이 없음의 오즈비가 독거노인이 비독거노인에 비해 1.47배 높았다 (95% CI 1.13-1.92). 동거인의 종류에 따라 분류하여 분석하였을 때, 흡연의 위험이 배우자 없이 젊은 세대와 함께 사는 가구의 노인은 배우자 및 젊은 세대와 함께 사는 노인에 비해 흡연의 위험이 9.12배(95% CI 2.04-40.80) 높았으며, 혼자 사는 노인도 1.41배 높았다(95% CI 1.03-1.92) (Table 3).

고찰

연구 결과, 남성의 흡연과 여성의 신체활동 없음의 위험이 독거노인에서 더 높았으며, 남성의 짜게 먹는 식습관과 여성의 흡연은 동거인의 유형에 따라 위험이 달라짐을 확인할 수 있었다.

Volume: 39, Article ID: e2017034

https://doi.org/10.4178/epih.e2017034

기존의 Zhang 등 의 노인을 대상으로 한 연구에서는 자녀와 사는 남성에서 흡연의 위험이 낮고, 자녀 또는 자녀와 배우자와 같이 사는 여성에서 흡연의 위험이 낮다고 보고하였다. 또한 남녀 모두 자녀와 배우자와 함께 사는 노인이 독거노인에 비해 음주의 위험이 낮다고 보고하였다. 우리 연구 결과에서는 남성의 경우에는 비독거 노인에서 흡연의 위험이 낮았고, 여성에서는 자녀와 배우자와 같이 사는 여성에서는 흡연의 위험이 낮았지만, 자녀와만 사는 경우에는 오히려 흡연의 위험이 높게 나타났다. 또한 음주에서는 독거와 동거인의 종류가 연관이 있지 않은 것으로 나타났다.[5] 기존의 연구에서는 가족과의 동거가 직접적 사회적 통제와 간접적 사회적 통제를 통해 건강습관에 영향을 미칠 것으로 설명하였다.[11] 직접적 사회적 통제란, 주변인의 설득, 요청 등을 통해 개인의 건강습관이 바뀌는 것을 말하며, 간접적 사회적 통제는 가족 구성원에 대한 책임감에 의해 개인이 보다 건강한 습관을 갖기 위해 애쓰게 되는 것을 말한다. 기존의 연구 결과와 우리 연구결과를 종합하여 생각할 때, 남성의 흡연에서는 동거자가 가하는 직간접적인 사회적 통제의 영향이 큰 것으로 생각된다. 여성의 경우, 한국의 현재의 노인세대에서 여성이 담배를 피우는 것은 오랫동안 사회에서 부적절하다고 인식되어 왔다.[12] 이런 사회에서 담배를 피우는 여성은 남성흡연자에 비해 흡연에 대한 애호가 더 강할 것으로 생각되며, 이와 같은 경우에는 더 많은 사람들이 사회적 통제를 가할 때에 효과가 나타난 것으로 생각된다. 더군다나 노인은 자녀들이 장성했으므로, 부모로서의 책임감과 의무감이 젊은 부모보다 덜하여, 부모의 역할로 인한 간접적 사회적 통제의 영향을 더 적게 받게 되

Volume: 39, Article ID: e2017034

https://doi.org/10.4178/epih.e2017034

므로, 우리 연구 결과에서 나타난 것처럼 자녀와만 사는 경우에도 흡연의 위험이 높게 나타날 수 있다.[13] Zhang 등 의 연구와 달리 우리 연구에서는 남녀모두 음주가 독거 및 동거인의 종류와 유의한 연관이 없는 것으로 나타났는데, 이것은 연구 환경의 문화차이에서 비롯될 가능성을 생각해 볼 수 있다.

Zhang 등 의 연구와 비교하여 우리 연구는 짜게 먹는 식습관과 신체 활동의 연관성도 조사하였다. 우리 연구에서는 여성노인에서 신체활동 없음의 위험이 독거노인에게서 유의하게 높다고 나타났다. 기존의 연구에서 신체활동에 대한 장애물로서 같이 운동할 사람의 부재를 대답한 여성 노인의 비율은 남성노인에 비해 2배 더 높았다.[14] 노인층에서 주로 같이 운동하는 사람이 가족과 친구라는 점을 고려할 때,[15] 여성 독거노인이 남성에 비해 같이 운동할 동료의 부재로 운동을 더 못하는 경우가 많을 것이라고 생각된다.

흥미롭게도 짜게 먹는 습관은 남성에서만, 노인 부부 가정에 속할 때에 통계적으로 유의하게 오히려 덜 짜게 먹는 것으로 나타났다. 이는 한국 사회에서 남성 노인은 거의 음식을 직접 만들어 먹지 않고, 다른 가족 구성원이 만든 음식을 먹는 것과 관련이 있을 것으로 생각된다. 노인 부부 가정에서는 부인, 기타 가정에서는 본인보다 어린 다른 가족 구성원이 만든 음식을 주로 먹을 것으로 생각되는데, 우리 자료원으로 통계 분석을 한 결과, 60-74세 여성 노인은 19세-59세의 성인에 비해 짜게 먹는 사람의 비율이 낮은 것으로 나타났다 (22.4% vs. 27.0%, p-value <0.01). 여성의 경우에는 노인 부부 가정에서도

Volume: 39, Article ID: e2017034

https://doi.org/10.4178/epih.e2017034

본인이 요리를 하고, 젊은 세대와 사는 경우에도 본인이 요리를 하는 경우가 많기 때문에, 남성처럼 가구유형에 따른 차이가 나타나지 않는 것으로 생각된다.

위와 같은 점들을 생각한다면, 독거여부 및 동거인의 종류가 건강행태에 미치는 영향이 성별에 따라 특이적으로 나타나며 이는 성별에 따라 생활 습관이 발전하는 과정이 다름에서 기인하므로, 노인의 건강행태 개선을 위한 정책을 만들 때에 이러한 점을 고려할 필요함을 알 수 있다. 남성의 경우 흡연의 위험이 높은 독거 노인에 대해 교육, 설득, 요청 등의 직접적 사회적 통제를 가하는 방법을 고려할 수 있을 것이다. 여성의 경우, 독거노인에서 신체활동의 위험이 낮은 것에 대해, 독거노인에게 운동 친구를 만들어주거나, 지역사회 단체운동활동 서비스 등을 제공하여 도움을 줄 수 있을 것이다.

이 연구는 몇 가지 제한점을 가지고 있다. 첫째, 선택 바이어스의 가능성이 있다. 지역사회건강조사는 1대1 면접조사로 진행되기 때문에, 3회 이상 접촉이 불가능하거나 강력하게 조사를 거부한 경우에는 조사를 진행할 수 없다. 따라서 조사를 진행하지 못한 대상자에게서 더 나쁜 건강행태를 가지고 있었을 가능성이 있다. 둘째, 결과 변수 중 짜게 먹는 습관은 객관화된 응답이 아니라, 본인이 스스로 생각한 주관적인 관점에 의한 응답이므로, 이에 따른 측정의 부정확성의 가능성이 있다. 그렇지만, 주관적인 응답과 실제 사이의 양의 연관성에서 유의한 차이는 크지 않을 것으로 생각된다. 셋째, 분석에서 혼란변수로서 사용한 고혈압, 당뇨, 뇌졸중, 심근경색의 진단은 건강상태의 지표이기 때문에, 우

리 논문의 결과 변수인 건강행태로부터 유래되는 2차적인 결과 변수가 될 수도 있다. 그러나, 이는 또한 건강행태에 영향을 미치는 중요한 원인으로써 빼 놓을 수 없다고 판단하였으므로, 이 분석에서는 건강행태의 결과보다 원인으로써의 역할이 더 크다고 생각하여 혼란변수로 두었다. 그러므로, 이 분석결과는 고혈압, 당뇨, 뇌졸중, 심근경색의 건강결과가 동일한 사람에서 분석한 결과로 볼 수 있다. 마지막으로, 이 연구는 기존의 연구와 마찬가지로 동거자의 존재뿐만 아니라 그들의 건강행태 속성이 다른 영향을 줄 수 있다는 점에 대해서는 연구하지 못했다. 이에 대해서는 앞으로 추가적인 연구가 필요할 것으로 생각된다.

결론

연구 결과 흡연은 남녀 모두 독거노인이 배우자, 젊은 세대와 함께 사는 가구의 노인에 비해 위험이 높으며, 여성에서는 배우자 없이 젊은 세대와 함께 사는 가구 또한 흡연의 위험이 높은 것으로 나타났다. 여성에서 신체활동 없음의 위험이 독거노인에서 더 높았다. 남성에서 짜게 먹는 습관의 오즈비는 노인 부부 가구에 속한 경우, 오히려 배우자 및 젊은 세대와 함께 사는 가구에 비해 낮은 것으로 나타났다. 그러나 음주에서는 독거 여부 및 동거인의 유형에 따른 유의한 차이가 나타나지 않았다. 그러므로 이와 같은 결과와 분석을 바탕으로 성특이적으로 나타난 노인의 건강행태를 개선하기 위해 적극적으로 생활 행태를 교정할 수 있도록 영향을 주려는 정책을 만들 수 있을 것이다.

Volume: 39, Article ID: e2017034

https://doi.org/10.4178/epih.e2017034

REFERENCES

1. Kim IK. Health innovation for aging society. Healthc Inform Res 2015;21:63-66.

2. Schone BS, Weinick RM. Health-related behaviors and the benefits of marriage for elderly persons. Gerontologist 1998;38:618-627.

3. Lewis MA, Butterfield RM. Social control in marital relationships: effect of one's partner on health behaviors. J Appl Soc Psychol 2007;37:298-319.

4. Umberson D. Gender, marital status and the social control of health behavior. Soc Sci Med 1992;34:907-917.

5. Zhang J, Wu L. Cigarette smoking and alcohol consumption among Chinese older adults: do living arrangements matter? Int J Environ Res Public Health 2015;12:2411-2436.

6. Kang YW, Ko YS, Kim YJ, Sung KM, Kim HJ, Choi HY, et al. Korea Community Health Survey data profiles. Osong Public Health Res Perspect 2015;6:211-217.

7. Kim H, Lee K, Chang S, Kang G, Tak Y, Lee M, et al. Factors affecting the validity of self-reported data on health services from the community health survey in Korea. Yonsei Med J 2013;54: 1040-1048.

8. Kim SJ, Han JA, Kim YH, Choi BY, Kim SY, Lee HJ, et al Test-retest reliability of health behavior items in the Community Health Survey in South Korea. Epidemiol Health 2015;37:e2015045.

9. Aaberge R, Melby I. The sensitivity of income inequality to choice of equivalence scales. Rev Income Wealth 1998;44:565569.

10. Ezzati M, Lopez AD, Rodgers A, Murray CJ. Comparative quantification of health risks: global and regional burden of disease attributable to selected major risk factors; 2004 [cited 2017 Aug 31]. Available from: http://apps.who.int/iris/bitstream/10665/42792/1/9241580348_eng_Volume1.pdf.

11. Umberson D. Family status and health behaviors: social control as a dimension of social integration. J Health Soc Behav 1987;28: 306-319.

12. Jung-Choi KH, Khang YH, Cho HJ. Hidden female smokers in Asia: a comparison of self-reported with cotinine-verified smoking prevalence rates in representative national data from an Asian population. Tob Control 2012;21:536-542.

13. Kim JG. The impact of family type on health behavior of elderly people. J Welf Aged 2011;51:35-56 (Korean).

Volume: 39, Article ID: e2017034

https://doi.org/10.4178/epih.e2017034

14. Moschny A, Platen P, Klaassen-Mielke R, Trampisch U, Hinrichs T. Barriers to physical activity in older adults in Germany: a crosssectional study. Int J Behav Nutr Phys Act 2011;8:121.

15. Böhm AW, Mielke GI, da Cruz MF, Ramirez VV, Wehrmesister FC. Social support and leisure-time physical activity among the elderly: a population-based study. J Phys Act Health 2016;13:599605.
